# G_i/o_ Protein-Dependent and -Independent Actions of Pertussis Toxin (PTX)

**DOI:** 10.3390/toxins3070884

**Published:** 2011-07-15

**Authors:** Supachoke Mangmool, Hitoshi Kurose

**Affiliations:** 1 Department of Pharmacology, Faculty of Pharmacy, Mahidol University, 447 Sri-Ayudhaya, Rajathevi, Bangkok 10400, Thailand; Email: pysmm@mahidol.ac.th; 2 Department of Pharmacology and Toxicology, Graduate School of Pharmaceutical Sciences, Kyushu University, 3-1-1 Maidashi, Higashi-ku, Fukuoka 812-8582, Japan

**Keywords:** A-protomer, ADP-ribosylation, B-oligomer, G_i/o_-dependent, G_i/o_-independent, heterotrimeric G protein, G protein-coupled receptor, pertussis toxin, Toll-like receptor 4

## Abstract

Pertussis toxin (PTX) is a typical A-B toxin. The A-protomer (S1 subunit) exhibits ADP-ribosyltransferase activity. The B-oligomer consists of four subunits (S2 to S5) and binds extracellular molecules that allow the toxin to enter the cells. The A-protomer ADP-ribosylates the α subunits of heterotrimeric G_i/o_ proteins, resulting in the receptors being uncoupled from the G_i/o_ proteins. The B-oligomer binds proteins expressed on the cell surface, such as Toll-like receptor 4, and activates an intracellular signal transduction cascade. Thus, PTX modifies cellular responses by at least two different signaling pathways; ADP-ribosylation of the Gα_i/o_ proteins by the A-protomer (G_i/o_ protein-dependent action) and the interaction of the B-oligomer with cell surface proteins (G_i/o_ protein-independent action).

## Abbreviations

AC: adenylyl cyclase
CHO: Chinese hamster ovary
CaMKII: calmodulin kinase II
CREB: cAMP response element-binding
8-CPT: 8-(4-chlorophenylthio)-2'-*O*-methyladenosine-3',5'-cyclic monophosphate
eEF2: eukaryotic elongation factor
Epac: exchange protein directly activated by cAMP
ERK: extracellular signal-regulated kinase
GPCRs: G protein-coupled receptors
G_i/o_PCRs: G_i/o_ protein-coupled receptors
GPIb: glycoprotein Ib
MAPK: mitogen-activated protein kinase
NAD^+^: nicotinamide adenine dinucleotide
PKA: cAMP-dependent protein kinase
PTX: Pertussis toxin
TLR4: Toll-like receptor 4
HUVECs: human umbilical vein endothelial cells
SAA: serum amyloid A
SPC: sphingosylphosphorylcholine
TCR: T-cell receptor

## 1. Introduction

Bacterial pathogens utilize their toxins to modify or kill host cells. The bacterial ADP-ribosylating toxins are a large family of dangerous and lethal toxins that include pertussis toxin (PTX), cholera toxin, diphtheria toxin, and pseudomonas exotoxin A [1,2]. These toxins are found in a diverse range of bacterial pathogens and are the cytotoxic agents that cause severe infectious diseases including whooping cough, cholera, and diphtheria (Table 1).

**Table 1:** Characteristics of ADP-ribosylating toxins from several virulent strains of bacteria.

ADP-Ribosylating Toxin	Bacterium	Target	Pathological Effect
Pertussis toxin	*Bordetella pertussis*	Cysteine residue of Gα_i_ subfamily (Gα_i_, Gα_o_, and Gα_t_) except Gα_z_	Gα_i_ protein-receptor coupling is inhibited, and its signal transduction is blocked.
Cholera toxin	*Vibrio cholerae*	Arginine residue of Gα_s_ subfamily (Gα_s_ and Gα_olf_)	As GTPase activity of the stimulatory Gα_s_ is inhibited, Gα_s_ protein is permanently activated.
Heat-labile enterotoxin	*Escherichia coli*	Arginine residue of Gα_s_ subfamily (Gα_s_ and Gα_olf_)	As GTPase activity of stimulatory Gα_s_ is inhibited, the Gα_s_ protein is permanently activated.
Diphtheria toxin	*Corynebacterium diphtheirae*	Diphthamide of eEF2	Protein synthesis is blocked.
Exotoxin A	*Pseudomonas aeruginosa*	Diphthamide of eEF2	Protein synthesis is blocked.

eEF2 = eukaryotic elongation factor 2.

Pertussis toxin (PTX) is the ADP-ribosylating toxin produced by the whooping cough causing bacterium *Bordetella pertussis* [3]. PTX catalyzes the ADP-ribosylation of the α subunits of the heterotrimeric G_i/o_ protein family (Gα_i_, Gα_o_, and Gα_t_; except Gα_z_), thereby preventing the G proteins from interacting with their cognate G protein-coupled receptors (GPCRs) [4]. ADP-ribosylation of the α subunit of heterotrimeric G_i/o_ proteins (Gα_i/o_) locks the α subunits into an inactive state (GDP-bound form), thus it is unable to inhibit adenylyl cyclase (AC). This modification of the Gα_i/o_ proteins results in the enhanced accumulation of cAMP, which is one of the mechanisms by which PTX induces the various pathological effects in host cells. 

PTX is composed of an A-protomer and B-oligomer. The A-protomer exerts ADP-ribosyltransferase activity on the Gα_i/o_ proteins, leading to inhibition of receptor-G protein coupling [5,6]. The B-oligomer of PTX recognizes and binds carbohydrate-containing receptors that deliver A-protomer into the cytosol [7]. Although many of the effects of PTX are dependent on ADP-ribosylation of the Gα_i/o_ proteins, G_i/o_ protein-independent effects of PTX have also been reported. For example, interaction of the B-oligomer with receptors on certain eukaryotic cells can mediate biological effects that are independent of the catalytic activity of A-protomer, including enhancement of immune responses [8,9,10], an increase in adenosine A1 receptor density [11], and the activation of tyrosine kinase, mitogen-activated protein kinase (MAPK), and NF-κB [12,13,14]. Moreover, we recently demonstrated a novel function of PTX that induces up-regulation of angiotensin II type 1 receptor independently of ADP-ribosylation of Gα_i/o_[15]. Thus, PTX can mediate biological effects through at least two signaling pathways; (1) G_i/o_ protein-dependent pathway through ADP ribosylation of the Gα_i/o_ proteins and (2) G_i/o_ protein-independent pathway by the binding of B-oligomer to cell surface proteins such as Toll-like receptor 4 (TLR4) [15] but not GPCRs.

In this paper, we review our current understanding of the G_i/o _ protein-dependent and G_i/o_ protein-independent pharmacological effects of PTX. 

## 2. Structure of Pertussis Toxin

The PTX molecule is a complex ADP-ribosylating toxin composed of five different subunits: S1, S2, S3, S4 and S5, presented in a ratio of 1:1:1:2:1 and arranged in the A-B architecture [16]. The A-protomer consists of a single S1 subunit that is responsible for ADP-ribosyltransferase activity [17], while the B-oligomer comprises S2, S3, S5, and two S4 subunits [18]. The A-protomer catalyzes the ADP-ribosylation of a cysteine residue in the α subunit of the heterotrimeric G_i/o_ protein subfamily, whereas the B-oligomer is responsible for binding to specific cell surface receptors and delivering the A-protomer into recipient cells [7].

**Figure 1:**
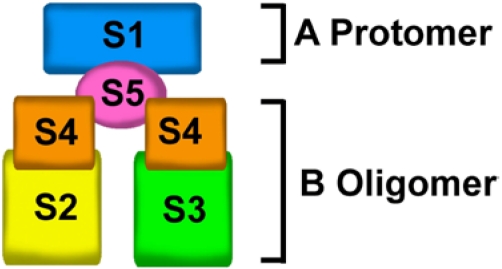
Pertussis toxin (PTX) structural organization. PTX contains five different subunits that are arranged in a typical A-B structure. The A-protomer contains an enzymatically active S1 subunit that is on the top of B-oligomer. The B-oligomer is composed of two dimers, S2-S4 and S3-S4 dimers, which are held together by the S5 subunit.

The crystal structure of PTX [18] revealed that the B-oligomer is composed of 5 noncovalently linked subunits which are organized as a triangular platform around a single catalytic S1 subunit that is on the top of the platform (Figure 1).

Exposing PTX to urea results in dissociation of the A-protomer (S1 subunit) from the B-oligomer and breakdown of the B-oligomer into three moieties: S2-S4 dimer, S3-S4 dimer, and S5 monomer [16]. These results suggest that the B-oligomer consists of two dimers, the S2-S4 and S3-S4 dimers, which are held together by the S5 subunit.

The S2 and S3 subunits of PTX share approximately 75% nucleotide and 70% amino acid homology [19,20]. Despite more than 75% sequence homology, structural and functional studies using site-directed mutagenesis of the S2 and S3 subunits have shown that the S2 subunit mediates binding to nonsialylated glycans, whereas S3 selectively binds sialylated oligosaccharides [21,22].

The S1 subunit of PTX contains regions of sequences homology to the catalytic portion of other ADP-ribosylating toxins such as the cholera toxin, enterotoxin, diphtheria toxin, and exotoxin A [1]. The carboxyl terminus of the S1 subunit is composed of 235 amino acid residues [23]. Previous studies have indicated that the carboxyl terminus is important in the interaction of the S1 subunit with the B-oligomer [24,25]. Residues 195 to 204 are required for optimal ADP-ribosylation of the α subunit of heterotrimeric G_i/o_ proteins. Residues 205 to 219 are linked to the catalytic region of S1 and are the B-oligomer-binding site of S1 subunit. Residues 220 to 235 are hydrophobic and are important for interaction of the S1 subunit with the B-oligomer [23].

The A protomer contains two cysteine residues at positions 41 and 201, which form disulfide bonds in the native PTX holotoxin. [26]. Reduction of this disulfide bond by dithiothreitol leads to a marked stimulation of S1 catalytic activity, which coincides with a release of the S1 subunit from B-oligomer [27]. Moreover, the presence of ATP leads to the dissociation of the S1 subunit from B-oligomer [28]. This dissociation makes the disulfide bond of the S1 subunit susceptible to cleavage by intracellular reducing compounds.

## 3. ADP-Ribosylation Mechanism of PTX

**Figure 2:**
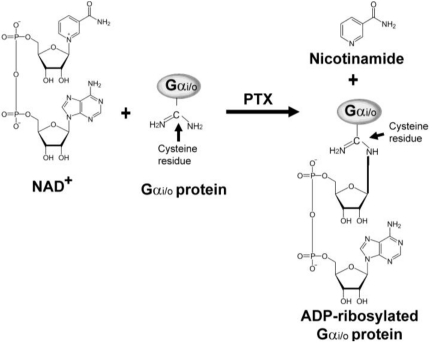
Schematic diagram of the ADP-ribosylation of α subunit of heterotrimeric G_i/o_ protein by pertussis toxin (PTX). PTX catalyzes the cleavage of the C-N bond between a carbon atom of ribose and a nitrogen atom of nicotinamide, and transfers the ADP-ribosyl moiety to an acceptor molecule.

Following attachment of PTX to host cells, S2 and S3 subunits of B-oligomer bind to the exposed sugar residues of glycolipid (gangliosides) on the plasma membrane of host cells. The A-protomer (S1 subunit) penetrates through the membrane and is released from B-oligomer into the cytoplasm. However, the exact molecular events associated with the entry of PTX into host cells are not fully understood. Once inside the cell the A-protomer ribosylates specific target proteins such as the α subunit of heterotrimeric G_i/o_ proteins (Figure 2) through its ADP-ribosyltransferase activity. ADP-ribosylation is also responsible for the actions of other bacterial ADP-ribosylating toxins, such as cholera toxin, diphtheria toxin, and exotoxin A.

PTX catalyzes the cleavage of the C-N bond between carbon atom of ribose and nitrogen atom of nicotinamide and transfer the ADP-ribosyl moiety from nicotinamide adenine dinucleotide (NAD^+^) to an acceptor molecule on the target protein (Figure 2). The target proteins for other ribosylating toxins include the eukaryotic elongation factor (eEF2) for diphtheria toxin [29] and arginine residue of the Gα_s_ protein for cholera toxin, which, like the Gα_i/o_-protein target for PTX, are involved in cell signal transduction [30]. In the case of PTX, ADP-ribosylation of the Gα_i/o_-proteins prevents the coupling to their cognate GPCRs and consequently disrupts the signal transduction cascade [31,32]. Besides their function as ADP-ribosyl transferases, the ADP-ribosylating toxins also have NAD^+^ glycohydrolase activity in the absence of an acceptor molecule [17]. However, this activity does not seem to contribute to any effects of PTX in the cell. 

**Figure 3:**
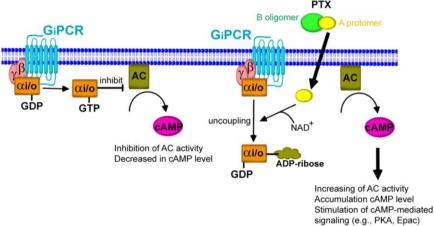
Uncoupling of Gα_i/o_ proteins from their cognate G protein-coupled receptor (GPCR). Activation of GPCRs leads to dissociation of heterotrimeric G protein complex into Gα_i/o_ and βγ subunit. The exchange of GTP from GDP results in activation of the inhibitory G protein (Gα_i/o_), thereby inhibiting adenylyl cyclase (AC) activity. When the A-protomer of PTX penetrates into the host cells, the Gα_i/o_ is ADP-ribosylated at cysteine residue resulting in inactivation of Gα_i/o_. The inhibitory effect of Gα_i/o_ on AC activity results in the elevation of intracellular cAMP levels, leading to activation of the cAMP-mediated signaling pathway. This enhanced pathway by PTX is recognized as the G_i/o_ protein-dependent pathway.

The amino acid ADP-ribosylated by PTX is cysteine, which is located four residues from the carboxyl terminus of the α subunits of the G_i/o_ proteins [33]. The uncoupling of GPCR from the Gα_i/o_ proteins results in disruption of the communication between receptor and the effector molecule AC. Thus, the Gα_i/o_ protein is inactivated and cannot perform its normal function to inhibit AC. In this way it prevents the signal from G_i/o_PCRs. Thus, the conversion of ATP to cAMP cannot be halted, resulting in excess intracellular cAMP level and the subsequent disruption of many cellular processes as shown in Figure 3. With the exception of Gα_z_, all members of the Gα_i/o_ protein family are substrates for PTX.

## 4. G_i/o_ Protein-Dependent Actions of PTX

The A-protomer (S1 subunit) of PTX catalyzes the ADP ribosylation of the membrane-bound regulatory G_i/o_ proteins resulting in inactivation, preventing them from inhibiting AC. As the inhibition of cAMP production by inhibitory GPCRs is only abolished by PTX, PTX treatment results in the enhancement of stimulatory GPCR-induced cAMP production. It is possible that the biological effects of PTX are a result of the disruption of the cAMP-mediated signaling pathway. As the G_i/o_ proteins are expressed in various tissues, the effects of PTX are observed in almost all cells. Some of the affected cells include the β-cells of pancreatic islets (hence the former name of PTX is islet-activating protein), adipocytes, macrophages, lymphocytes, and other cells that cause paroxysms and neurological disturbance. The ADP-ribosylation of the G_i/o_ proteins by PTX is irreversible. As a result, restoration of G_i/o_ function in the cells is dependent on G_i/o_ protein replacement of ADP-ribosylated G_i/o_ proteins with new proteins. Unlike cholera toxin, which acts to inhibit GTPase activity of the G_s_ protein, PTX does not affect basal GTPase activity of the G_i/o_ proteins, only receptor-stimulated GTPase.

The biological effects of PTX that result from ADP-ribosylation of the Gα_i/o_ proteins are very diverse, some of which can be attributed to the ADP-ribosylation of the α subunits of the heterotrimeric G_i/o_ protein family, as shown in Table 2. 

**Table 2:** Gα_i/o_ protein family.

α Subunit	Amino Acid	Expression [34,35]	Effect on Effectors	Toxin (Site of Action)
α_o_	354	Heart, neurons, neuroendocrine cells	Inhibition of AC activity [36]	PTX at cysteine 351 [1,37,38]
			Inhibition of Ca^2+^ channel [39]	
			Activation of K^+^ channel [40]	
α_i1_-α_i3_	354	Neurons and ubiquitous	Inhibition of AC activity [41]	PTX at cysteine 351 [1,37,38]
			Inhibition of Ca^2+^ channel [39,42]	
			Activation of K^+^ channel [40,43]	
α_z_	355	Platelets	Inhibition of AC activity [44]	-(cyteine modified by PTX does not present) [46]
			Inhibition of Ca^2+^ channel [45]	
			Activation of K^+^ channel [40]	
α_t_	350	Rod/cone outer segments	Activation of cGMP-PDE [47]	PTX at cysteine 351 [37,38]
				CTX at arginine147 [37]
α_gust_	353	Taste buds; sweet and/or bitter	Activation of cGMP-PDE [48]	PTX at cysteine 350 [37,38]

AC: adenylyl cyclase; PDE: phosphodiesterase.

Pancreatic cells show increased insulin secretion upon treatment with PTX [37]. Moreover, PTX inhibits insulin-stimulated autophosphorylation of insulin receptor by inactivation of insulin receptor kinase. Treatment of cells with the catalytically inactive B-oligomer had no effect on insulin receptor kinase activity [49]. These results suggest that PTX can modulate insulin receptor signaling at receptor level. The lipolytic action of PTX has been used as a sensitive assay for the action of toxin, and is caused by PTX-catalyzed ADP ribosylation of the Gα_i_ proteins in adipocytes. Adenosine is produced *in vitro* as one of the ATP degradation products during preparation of adipocytes from fat pads by the collagenase digestion method, and adenosine inhibits AC via the Gα_i_[2,50]. As lipolysis is enhanced by the increase in intracellular cAMP, inhibition of adenosine receptor-G_i/o_ coupling by PTX results in enhanced cAMP accumulation, leading to increased lipolysis.

*In* *vivo* administration of PTX induces lymphocytosis, a hallmark of systemic pertussis in children. It also causes hyperinsulinemia and hypoglycemia, as well as modification of histamine sensitization [2]. In addition, PTX, but not a noncatalytic mutant of PTX, inhibits neutrophil migration to rat peritoneal cavities in response to lipopolysaccharide [51]. The increase in skin vascular permeability induced by leukotriene B4 is also inhibited by PTX only, not a catalytically inactive mutant. These results suggest that PTX inhibits neutrophil migration and vascular permeability *in vivo* and these effects are dependent on the ADP-ribosyltransferase activity of the A-protomer.

Ligand binding to inhibitory GPCRs leads to activation of the G_i/o_ proteins and then causes the dissociation of heterotrimeric G_i/o_ proteins into an activated α_i/o_ subunit and a βγ subunit. The activated Gα_i/o_ subunits (GTP-bound form) inhibit AC activity, resulting in the decrease of intracellular cAMP level [30]. This attenuates the cAMP-mediated signaling pathway. Treatment of NG108-15 cells (neuroblastoma-glioma hybrid cells) with PTX attenuates receptor-mediated inhibition of AC, resulting in inhibition of the opiate-stimulated GTPase activity. Moreover, this inhibition effect by PTX requires NAD^+^, emphasizing that this effect of PTX is mediated by ADP- ribosylation of the Gα_i/o_ proteins. The Gα_i/o_ proteins are ADP-ribosylated by A-protomer, inhibiting the GDP-GTP exchange of the G_i/o_ proteins by GPCRs. The α subunit is locked in the inactive GDP-bound state resulting in the enhanced increase in cAMP level by stimulatory GPCRs. Thus, G_i/o_ protein-dependent effects of PTX are mainly mediated by cAMP action. Cyclic AMP is formed from ATP by the action of AC and degraded to AMP by cAMP phosphodiesterase [52]. Most effects of cAMP in animal cells are mediated by the action of cAMP-dependent protein kinase (PKA). The inactive form of PKA is a tetramer consisting of two catalytic and two regulatory subunits. The binding of cAMP to the regulatory subunits leads to their dissociation from the catalytic subunits. The free catalytic subunits are then active and able to phosphorylate serine and threonine residues of their target proteins. The free catalytic subunit of PKA also translocates to the nucleus and phosphorylates the transcriptional factor CREB (cAMP response element-binding), leading to the activation of cAMP-inducible genes [53]. Such regulation of gene expression by cAMP plays important roles in controlling the proliferation, survival, and differentiation of a wide variety of animal cells. Previous studies have identified a family of molecules known as Epac (exchange protein directly activated by cAMP) that directly bind cAMP and exhibit guanine nucleotide exchange factor activity toward Rap1 [54,55]. The binding of cAMP or the selective cAMP analogue 8-CPT (8-(4-chlorophenylthio)-2'-O-methyladenosine-3',5'-cyclic monophosphate) to Epac leads to activation of calmodulin kinase II (CaMKII)-dependent signaling pathways including the release of Ca^2+^ from sarcoplasmic reticulum [56] and the increased phosphorylation of phospholamban [57]. However, whether the increase in cAMP level by PTX-catalyzed ADP-ribosylation of the Gα_i/o_ proteins activates this Epac-mediated CaMKII signaling pathway remains to be elucidated.

## 5. G_i/o_ Protein-Independent Actions of PTX

ADP-ribosylation of the Gα_i/o_ proteins does not account for all responses by the interactions of PTX with host cells. PTX is reported to be a T-cell mitogen [7]. This action can be reproduced by B-oligomer alone suggesting that B-oligomer itself may induce the mitogenic action independent of A-protomer-induced ADP-ribosylation of the G_i/o_ proteins. Several other G_i/o_ protein independent actions of PTX have been reported, such as enhancement of immune responses [8,9,10], increase in adenosine A1 receptor density [11], and activation of tyrosine kinase, MAPK, and NF-κB [12,13,14]. Recently, we demonstrated that the B-oligomer of PTX induces Rac activation through a pathway independent of ADP-ribosylation of the Gα_i/o_ proteins [15]. PTX increases IL-1β induction through sequential activation of TLR4, Rac, NADPH oxidase, and NF-κB, which leads to up-regulation of angiotensin II type 1 receptor. Thus, PTX binds to two binding sites; one is TLR4 which activates Rac, and another is the binding site that is required for the entry of PTX into the cells to ADP-ribosylate the Gα_i/o_[15].

Interestingly, both the S1 mutant of PTX and the B-oligomer completely reproduced several effects of native PTX (holotoxin) action. For example, PTX-induced extracellular signal-regulated kinase (ERK) activation of endothelial cell is entirely independent of ADP-ribosylation of the Gα_i/o_[58]. However, inactivation of Gα_i_ proteins by PTX inhibits serum amyloid A (SAA)-mediated ERK activation in human umbilical vein endothelial cells (HUVECs) [59]. Preincubation of HUVECs in PTX (100 ng/mL) prior to sphingosylphosphorylcholine (SPC) stimulation markedly reduces the levels of phosphorylated MAPKs including ERK, indicating that Gα_i_ proteins have a role in SPC-induced ERK activity [60]. Thus it is not clear what causes the difference in activation *versus* inhibition of ERK activity. One possibility could be derived from the differential cellular mechanisms which regulate ERK activity.

Moreover, the B-oligomer alone was as effective as PTX in inducing glucose oxidation in rat adipocytes [7]. It was also reported that the binding of the B-oligomer to eukaryotic cells can alter cellular function independently of ADP-ribosylation. For example, PTX has been shown to bind to glycoprotein Ib (GPIb) on human platelets, leading to subsequent platelet aggregation [61]. The purified B-oligomer also induces mitogenic stimulation of human T cells [62,63], enhances glucose oxidation in adipocytes [64], induces dendritic cell maturation in a TLR4-dependent manner [14], and associates with T-cell receptor complex to initiate signal transduction in T-lymphocytes [65].

Upon prolonged incubation (at least 1 or 2 h) with PTX, the A-protomer is internalized by certain cells and subsequently activated by cleavage of the disulfide bond. The A-protomer then catalyzes the transfer of an ADP-ribose moiety from endogenous NAD^+^ to a carboxyl terminal cysteine residue in the Gα_i/o_ proteins (Figure 2). A previous study reported that PTX completely ADP-ribosylated the Gα_i/o_ proteins at 10 ng/mL within 3 h in Chinese hamster ovary (CHO) cells [66]. ADP-ribosylation of the G_i/o_ proteins disrupts their interaction with various inhibitory GPCRs, leading to blockade of certain transmembrane signaling process and eventually cellular intoxication [67]. In addition to its delayed inhibitory effect on the G_i/o_ proteins, PTX has also been shown to elicit rapid responses (in minutes) in a variety of cell types [64], which may have profound pathological effects as important as its ADP-ribosylation activity. PTX was shown to bind to human platelet GPIb, inducing rapid platelet aggregation and an increase of intracellular Ca^2+^ level [61,68]. Recent studies also reported that the B-oligomer interacts with extracellular receptors, such as the T-cell receptor (TCR), and activates an intracellular signal transduction pathway by inducing and interacting with the T-cell receptor complex [65,69]. In contrast to the rapid and transient effects caused by B-oligomer, the A-protomer (catalytic S1 subunit) must penetrate into the cytoplasm of host cells to ADP-ribosylate and inactivate the Gα_i/o_ proteins. This process can usually take hours, but permanently modifies G proteins [65,70]. It indicates that PTX-induced responses in T-cells can be divided into two phases: short-term by the B-oligomer, and long-term by the catalytic A-protomer. From these data, it has been concluded that G_i/o_ protein-dependent effects of PTX through ADP-ribosylation occur more slowly than G_i/o_ protein-independent effects of PTX through the B-oligomer (Table 3). 

PTX detoxified by formaldehyde treatment or genetic modification maintains its protein structure and the immunological properties, but not its enzymatic activity [71,72]. This detoxified PTX is able to activate TLR4 in monocyte derived dendritic cells, a property previously reported for fully active PTX and its B-oligomer [14]. A low dose of detoxified PTX efficiently triggers TLR4 signaling, while a high dose is necessary to activate both TLR4 and TLR2 [73]. This is the first report showing that detoxified PTX also triggers TLR2-mediated signal transduction pathway. As TLR4 plays an essential role in expansion of Th1/Th17 immunity, detoxified PTX may work as an alternative adjuvant to promote Th1/Th17 responses. 

Interestingly, a previous study showed that while both PTX and the B-oligomer induce dendritic cell maturation, the induction of cytokines that are produced differ [14]. However, the effect of both PTX and its B-oligomer on dendritic cell maturation was dependent on TLR4 [74]. It is possible that the differential cytokine production of dendritic cells by PTX and the B-oligomer are due to the differences in their utilization of TLR4-mediated intracellular signaling pathways, which may be accounted for by both ADP-ribosylation-dependent and -independent responses.

The toxic effects mediated by catalytic activity of the native PTX (holotoxin) occurs at very low toxin concentrations and appears to alter the function of tissues and organs very distal from the site of bacterial growth. In contrast, effects of PTX mediated by interaction of the B-oligomer with many cell surface proteins require higher doses of the toxin [65]. For example, the B-oligomer of PTX caused concentration-dependent platelet activation, as determined by increasing intracellular Ca^2+^ concentration and dense granule secretion [61]. In addition to its role in delivering the S1 subunit into the cells, the B-oligomer elicits biological effects such as an increase in intracellular Ca^2+^, activation of T-lymphocytes [62,75] and platelet aggregation [68]. These B-oligomer-mediated effects may be elicited by the binding of the B-oligomer to a specific receptor and require significantly higher concentrations of toxin than are necessary for the ADP-ribosyltransferase activity of the A-protomer.

Moreover, it appears that most cells would be susceptible to PTX as a result of ADP-ribosylation of the Gα_i/o_ proteins and that PTX holotoxin-mediated effects could occur at many sites in host cells [72]. Recent findings demonstrate that the G_i/o_ protein-dependent action of PTX (via ADP-ribosylation of Gα_i/o_) exhibits the differences in signaling pathway when compare to that of G_i/o_ protein-independent action of PTX (via B-oligomer) as summarized in Table 3.

**Table 3:** The differences in characteristics of G_i/o _ protein-dependent and -independent effects of PTX.

	G_i/o_ Protein-Dependent Effects of PTX	G_i/o_ Protein-Independent Effects of PTX
Subunit	A-protomer (S1 subunit)	B-oligomer (S2-S3 dimer, S2-S5 dimer, and S5 monomer)
Onset of action	Slow	Rapid
Concentration of toxin to induce the effects	Low	High
Biological effects	Enhance insulin secretion [49]	Induce dendritic cell maturation [14]
	Inhibit lymphocyte and neutrophil migration [52,76]	Inhibit growth cone guidance [78]
	Inhibit enkephalin stimulation of GTPase [77]	Induce myelomonocytic cell adhesion [79]
	Inhibit autophosphorylation and activation of insulin receptor kinase [50]	Induce ERK1/2 activation in endothelial cell [59]
		Activate platelet aggregation [62,69]
		Activate T lymphocyte [66,80]
		Induce Th1/Th17 immune response through MAPK and IL-10 [74]
		Activate tyrosine kinase signaling [12]
		Inhibit Tat-induced TGF- β production [81]
		Inhibit HIV type 1 replication [82]

## 6. Conclusions

**Figure 4:**
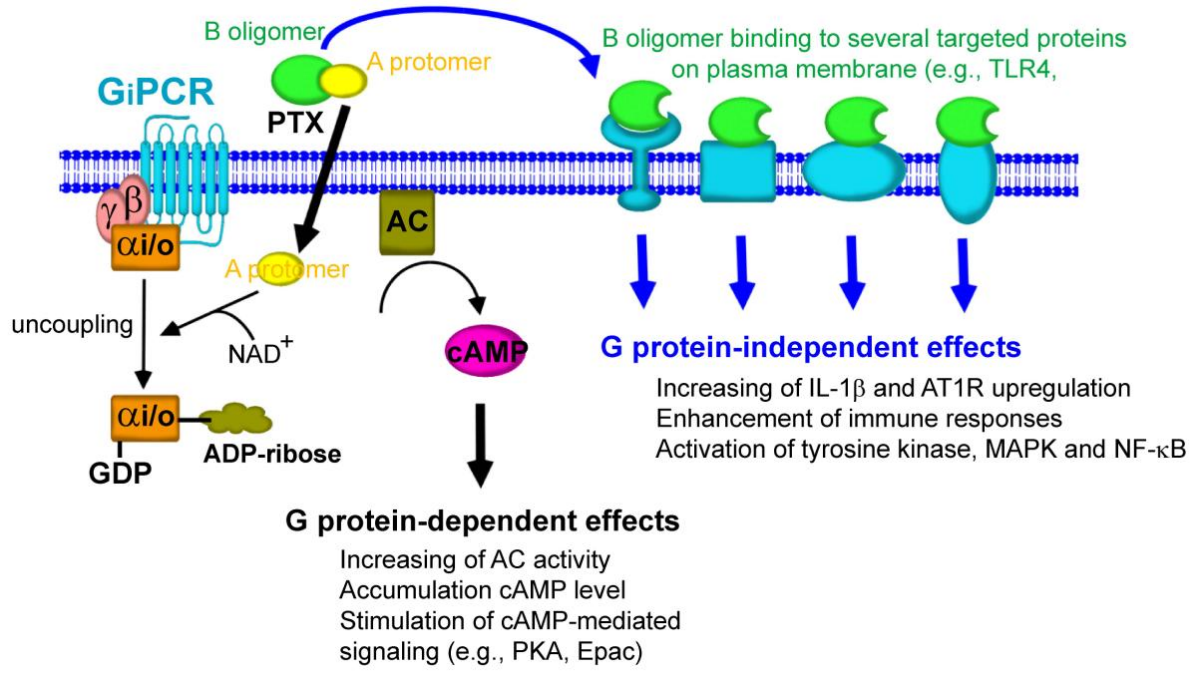
G_i/o_ protein-dependent and -independent effects of PTX. Following the binding of PTX to host cells, the A-protomer penetrates through the cell membrane. A-protomer is dissociated from B-oligomer and released into the cytoplasm. A-protomer then catalyzes the ADP-ribosylation of Gα_i/o_ that leads to uncoupling of Gα_i/o_ from its cognate inhibitory GPCRs. The inhibitory effect of Gα_i/o_ on AC activity is then halted, resulting in accumulation of cAMP. This action of PTX results in disruption of cellular function through cAMP-mediated signaling pathway (G_i/o_ protein-dependent effects). In a separate pathway, the B-oligomer binds to and interacts with several targeted proteins on the plasma membrane, leading to the induction of the biological responses that are independent of ADP-ribosylation of Gα_i/o_ protein (G_i/o_ protein-independent effects).

It is clear that PTX exhibits its various effects by eliciting at least two different signaling pathways (Figure 4). One involves the mobilization of the enzymatic A-protomer into host cells leading to the ADP-ribosylation of the α subunit of heterotrimeric G_i/o_ proteins [72]. This process called G_i/o_ protein-dependent effect of PTX, has a slow onset (at least 1-2 h) and requires a low concentration of the toxin. A second involves binding of the B-oligomer of PTX to several targeted proteins expressed on the cell surface such as TLR4 [15], GPIb [61], and TCR4 [65] initiating a series of rapid signaling events that require a higher concentration of the toxin. Thus, the B-oligomer of PTX appears to activate G_i/o_ protein-independent signaling in a diverse array of cell types.

It remains to be determined whether the binding site of PTX for the B-oligomer-mediated responses is the same as that of PTX to enter the cells for the A-protomer to ADP-ribosylate the G_i/o_ proteins. Another concern generated from these findings is whether the B-oligomer-mediated early transmembrane signaling events will influence the subsequent ADP-ribosylation of the Gα_i/o_ proteins. The various cellular responses of PTX cannot be concluded simply by modification of the G_i/o_ proteins, as the B-oligomer of PTX can also elicit cellular responses.

In this review, we summarize that PTX can mediate biological responses by at least two different signaling pathways including (1) G_i/o_ protein-dependent action of PTX through ADP-ribosylation of the Gα_i/o_ proteins and (2) G_i/o_ protein-independent action of PTX through the binding of the B-oligomer with cell surface specific proteins (Figure 4).
